# Dopamine D4 Receptor Agonist Drastically Increases Delta Activity in the Thalamic Nucleus Reuniens: Potential Role in Communication between Prefrontal Cortex and Hippocampus

**DOI:** 10.3390/ijms242015289

**Published:** 2023-10-18

**Authors:** J. Kuang, V. Kafetzopoulos, Richard Deth, B. Kocsis

**Affiliations:** 1 Department of Psychiatry, Beth Israel Deaconess Medical Center, Harvard Medical School, Boston, MA 02215, USA; junyi.kuang@gmail.com (J.K.); vkafetzop@med.uoa.gr (V.K.); 2 Department of Psychiatry, Medical School, University of Ioannina, 45110 Ioannina, Greece; 3 Department of Pharmaceutical Sciences, Nova Southeastern University, Fort Lauderdale, FL 33328, USA; rdeth@nova.edu

**Keywords:** hippocampus, prefrontal cortex, oscillatory coupling, delta rhythm, theta rhythm, cortical synchronization, dopamine 4 receptors, A-412997

## Abstract

Network oscillations are essential for all cognitive functions. Oscillatory deficits are well established in psychiatric diseases and are recapitulated in animal models. They are significantly and specifically affected by pharmacological interventions using psychoactive compounds. Dopamine D4 receptor (D4R) activation was shown to enhance gamma rhythm in freely moving rats and to specifically affect slow delta and theta oscillations in the urethane-anesthetized rat model. The goal of this study was to test the effect of D4R activation on slow network oscillations at delta and theta frequencies during wake states, potentially supporting enhanced functional connectivity during dopamine-induced attention and cognitive processing. Network activity was recorded in the prefrontal cortex (PFC), hippocampus (HC) and nucleus reuniens (RE) in control conditions and after injecting the D4R agonist A-412997 (3 and 5 mg/kg; systemic administration). We found that A-412997 elicited a lasting (~40 min) wake state and drastically enhanced narrow-band delta oscillations in the PFC and RE in a dose-dependent manner. It also preferentially enhanced delta synchrony over theta coupling within the PFC-RE-HC circuit, strongly strengthening PFC-RE coupling. Thus, our findings indicate that the D4R may contribute to cognitive processes, at least in part, through acting on wake delta oscillations and that the RE, providing an essential link between the PFC and HC, plays a prominent role in this mechanism.

## 1. Introduction

Network oscillations are essential for all cognitive functions. Oscillatory deficits are well established in psychiatric diseases and are recapitulated in animal models [1,2,3,4,5,6,7,8,9,10]. They are significantly and specifically affected by pharmacological interventions using psychoactive compounds [11,12,13,14,15,16]. The goal of this study was to test the effect of the activation of dopamine D4 receptors (D4Rs) on slow network oscillations at delta and theta frequencies in the prefrontal cortex (PFC) and hippocampus (HC) during wake states, potentially supporting enhanced functional connectivity during dopamine-induced attention and cognitive processing. D4R activation inhibits melatonin synthesis while increasing functional gamma activity during attention [14,17,18], promoting an overall state of wakeful alertness. Enhanced oscillatory activity could serve to promote the influence of this circuit in the PFC, linking theta-encoded contextual memory in the HC with current sensory experience. In this regard, the seven-repeat variant of the D4R is associated with novelty-seeking behaviors [19,20], implying a role in memory-based discrimination between novel and familiar experiences. The seven-repeat variant is also a well-established risk factor for attention-deficit hyperactivity disorder (ADHD) [21,22] and exhibits weaker signaling activity, including the inhibition of cAMP production [23], glutamate receptor regulation and the methylation of membrane phospholipids [24], which is a unique feature of the D4R [25]. Dysfunctional D4R-mediated methylation has been proposed as an underlying contributor to autism spectrum disorder [26]. 

In both the PFC and HC, aberrant gamma oscillations induced via D4R activation have implicated the interaction of parvalbumin-expressing interneurons and pyramidal neurons, landmark neurobiological features in various schizophrenia endophenotypes [27,28]. Enhanced gamma power induced via D4R activation was reported in a computational network model [29], in a brain slice [17,18], and in vivo in freely moving rats [14]. In the latter study, the systemic administration of the D4R agonist A-412997 at a 10 mg/kg dose induced enhanced abnormal gamma oscillations, dominating all recordings in the PFC and HC. The effect was dose-dependent; a not-overwhelming but significant gamma power increase was also demonstrated at 3 mg/kg but not at lower doses (0.3 and 1.0 mg/kg). D4R activation was also shown to increase delta power in the PFC and decrease theta in the HC in urethane-anesthetized rats [30], in which gamma oscillations were suppressed and thus slow rhythms were unmasked, even after 10 mg/kg of A-412997. Significant effects of 5 mg/kg injections on delta power were also shown, although in a relatively small sample. Thus, in this study, we used 3 and 5 mg/kg injections, aiming at a range that is effective for gamma enhancement but avoiding abnormally high gamma levels which would dominate all network activities and electrophysiological signals.

The second goal of this study was to test the potential effect of the D4R on the balance of frequency-tagged bidirectional relationships [2,31,32,33,34,35] between the PFC and HC in freely moving rats by recording from the thalamic nucleus reuniens (RE), which provides an anatomical link between the two structures in both directions, as opposed to the unidirectional HC-to-PFC monosynaptic pathway [36,37,38,39,40] There are several papers that have examined PFC-HC interactions [41,42] and also their disruption in animal models of schizophrenia [43,44]. The RE was previously shown to play a significant role in psychiatric diseases, including schizophrenia and major depression [45]. The HC-PFC pathway is monosynaptic with respect to HC-PFC communication. Conversely, the reverse pathway is mainly relayed via the RE. Thus, together, they provide a reciprocal circuit linking memory and executive functions. 

The impairment of delta and theta oscillations and potential disturbances in the delta–theta balance were implicated in schizophrenia [46], and the RE may play a role in maintaining this balance. The RE was shown to primarily convey PFC-delta to the HC [47], but due to its reciprocal projections with both the PFC and HC [36,37,38], it might as well participate in theta coupling. As the direct coupling of fast oscillations between distant structures is limited, their synchronous modulation via slow delta and theta rhythms is essential; their functional significance was widely demonstrated [2,46,48,49,50,51]. Directly relevant to the goals of the current study, a functional difference between delta and theta PFC-HC coupling, organized and mediated by the RE, clearly appeared in a recent study [34] focusing on the involvement of synchronizing memory networks at beta frequencies. Similar to gamma (~40 Hz) [2], PFC-HC beta (15–30 Hz) coherence is modulated by both delta and theta waves. Yajachandran et al. [34] recently reported that beta bursts dominating PFC-HC synchrony were delta-modulated, specifically in non-spatial memory tasks. 

We hypothesized that D4R activation will strengthen delta oscillations in the PFC-RE-HC network in freely moving rats, as in the urethane-anesthetized model [30]. If so, this mechanism might be involved in cognitive behaviors, e.g., in episodic-memory-based decision making, as addressed by Yajachandran et al. [34]. Enhanced coherence in the PFC-RE-HC circuit may also be a mechanism of D4R-related novelty-seeking behaviors which are potentially reduced in ADHD in conjunction with reduced D4R signaling.

## 2. Results

### 2.1. Delta Oscillations during Waking 

**Figure 1:**
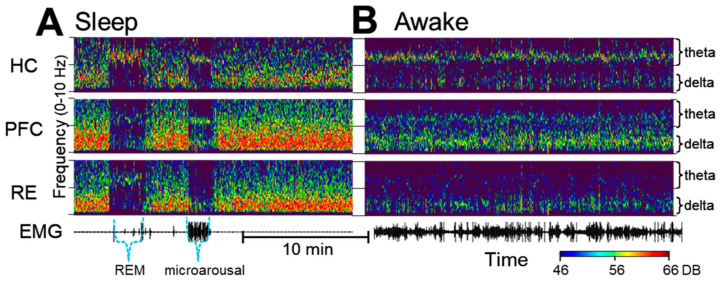
Delta and theta oscillations during sleep and waking under control conditions. (**A**). Time–frequency plots show wide-band delta activity covering the entire 1–4 Hz range during slow-wave sleep, interrupted by short episodes of rapid-eye-movement (REM) sleep and microarousals characterized by narrow band oscillations in the theta (~7 Hz) and in the delta (~2 Hz) bands. Note the theta in all three signals during REM sleep and the narrow-band delta rhythm in the prefrontal cortex (PFC) and the thalamic nucleus reuniens (RE) along with PFC and HC theta in a state of microarousal. (**B**). In waking, locomotor activity (see electromyogram, EMG) is accompanied by theta oscillations which are the strongest in the hippocampus (HC) but are also present in the PFC, coinciding with narrow-band delta oscillations which are strongest in the PFC but are present in the HC and in RE as well.

Delta activity in waking is fundamentally different from delta in SWS. Figure 1 shows the major distinctions between the two patterns of activity in sample recordings of the PFC, HC and RE in different sleep–wake states. Delta activity during SWS, which is a defining feature of this state, is not a regular oscillation. It is composed of slow waves that constantly vary in their length which are converted in the spectral space into a wide-band signal limited to the 1–4 Hz band. In contrast, real narrow-band oscillations, represented in this frequency range by sharp peaks in the power spectra, appear in waking and during motor activity, coincident with theta oscillations in the HC. Delta oscillations are primarily generated by networks in the frontal cortex, including the PFC, but both HC-theta and frontal delta waves are present widely and serve as powerful mechanisms of coupling between forebrain regions in a behavior-dependent manner (see e.g., [52]). Accordingly, the strength and frequency of narrow-band delta oscillations may vary over time, as shown in the 25 min recording in Figure 1B. As D4R agonists induce active waking [14], appropriate control segments with high EMG activity and prominent HC-theta were selected for a comparison with the electrical activity of the PFC, HC and RE networks after D4R activation. 

### 2.2. The Effect of Dopamine D4 Receptor Activation on Delta Activity in the PFC, HC, and RE

**Figure 2:**
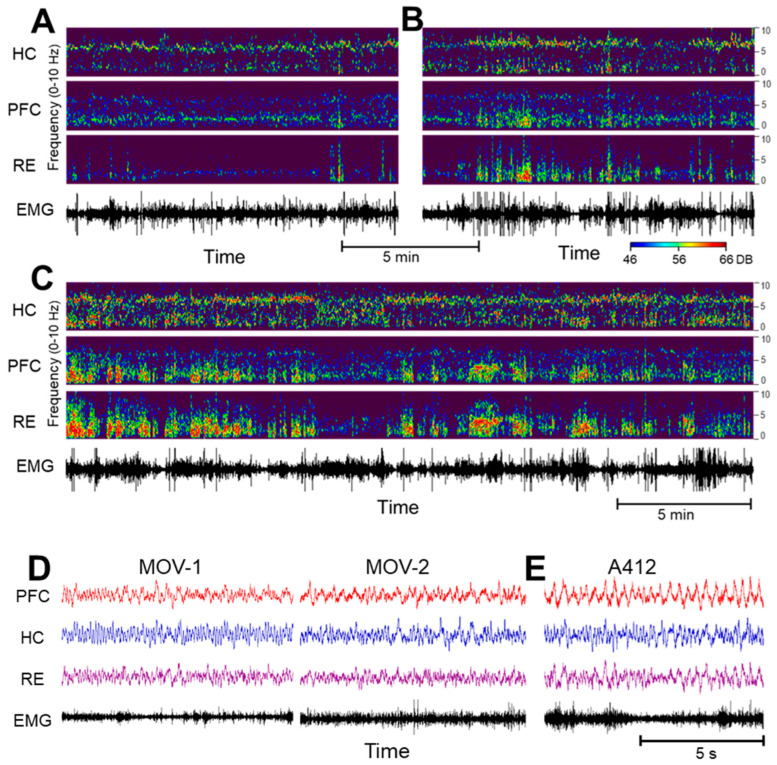
The dopamine 4 receptor (D4R) agonist’s effect on the activity of the hippocampus (HC), prefrontal cortex (PFC) and thalamic nucleus reuniens (RE). (**A**–**C**). Time–frequency plots of network activity in two control segments, showing natural variations in delta oscillations (**A**,**B**) and a strong increase in delta coinciding with theta in the HC after an injection of A-412997 (**C**) in a representative experiment. (Power scales (46–66 DB) are identical in all figures and are set to match the strongest signal peaks in (**C**)). (**D**,**E**). Traces (voltages) on an extended time scale in two control segments of active waking ((**D**), MOV-1 and MOV-2; note the electromyogram (EMG) activity and the dominant theta in the HC and RE) and after the A-412977 injection (**E**); note the large delta waves in the PFC and RE).

Figure 2 shows changes in low-frequency oscillations in the HC, PFC and RE after the D4R agonist A-412977 was injected at a dose of 5 mg/kg dose (i/p) in a representative experiment. To illustrate normal variations in theta and delta patterns in active waking, we show two samples of control signals selected from the same experiment, with weaker but stable delta oscillations in Figure 2A and stronger and more bursty delta oscillations in Figure 2B. After the injection, delta oscillations were drastically enhanced in all recordings. Traces of the original signals also indicate that the delta oscillations became more regular on a short time scale (Figure 2E). 

**Figure 3:**
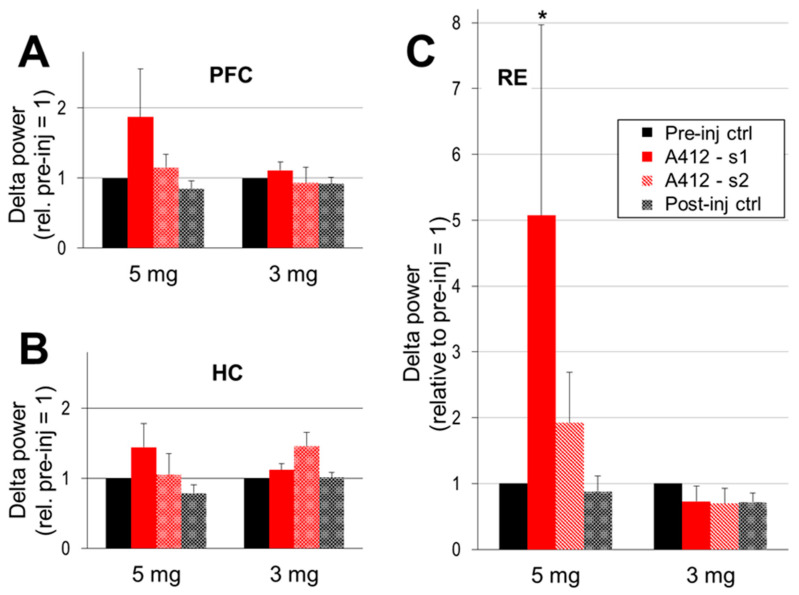
The Dopamine 4 receptor (D4R) agonist’s effect on cortical delta activity. Delta power (peak between 1–4 Hz, AVE and SEM) in the prefrontal cortex (PFC; (**A**)), hippocampus (HC; (**B**)) and thalamic nucleus reuniens (RE; (**C**)) in pre- and post-injection control segments and in the two consecutive segments after the injection, early (A412-s1; immediately following the injection) and late (A412-s2; lasting until the first signs of wake to sleep transition). The peak delta power was normalized in each experiment to the level of the control delta activity (i.e., the segments pre-inj ctrl = 1 in each individual experiment) and averaged over the group. A-412997 was injected at two doses, 5 mg/kg (*n* = 5) and 3 mg/kg (*n* = 6). Error bars show SEM values and asterisks show significant changes compared with the control (* *p* < 0.05; two-tailed Student’s *t*-test).

**Figure 4:**
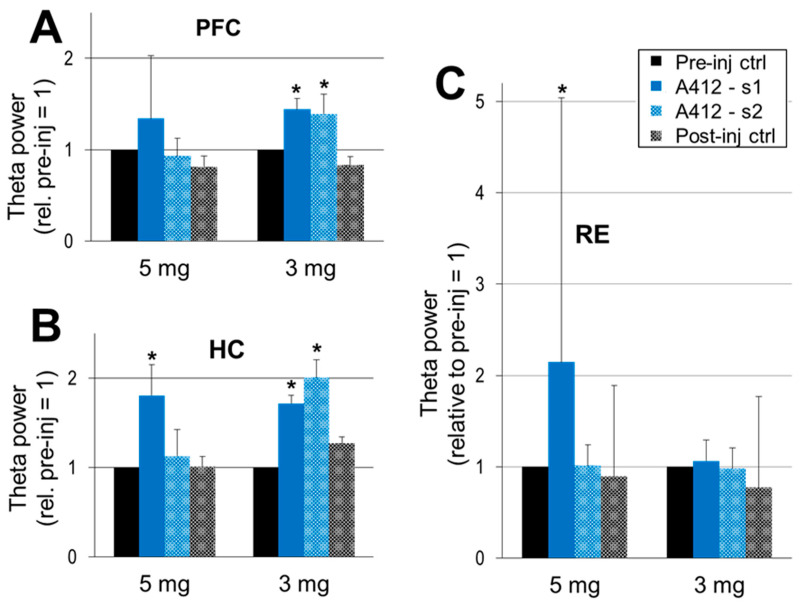
The dopamine 4 receptor (D4R) agonist’s effect on theta activity. Theta power in the prefrontal cortex (PFC) (**A**), hippocampus (HC) (**B**) and the thalamic nucleus reuniens (RE) (**C**) within the 5–10 Hz frequency range, normalized in each experiment to the level of theta activity in the control segments (pre-inj ctrl = 1 in each individual experiment) and averaged over the group. A-412997 was injected at two doses, 5 mg/kg (*n* = 5) and 3 mg/kg (*n* = 6). Error bars show SEM values, and asterisks show significant changes compared with control (* *p* < 0.05; two-tailed Student’s *t*-test).

**Figure 5:**
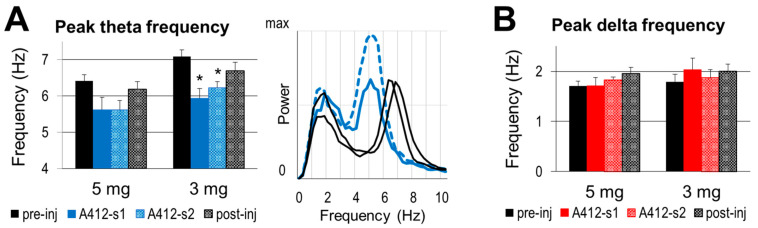
The dopamine 4 receptor (D4R) agonist’s effect on the frequency of slow oscillations. (**A**). Group averages of peak theta frequency in control segments and after the injection of A412997 in 5 and 3 mg/kg doses and sample autospectra (right) from one experiment under control conditions (black) and after an injection (blue) (**B**). Group averages of the peak delta frequency in the control segments and after the injection of A412997 in 5 (*n* = 5) and 3 mg/kg (*n* = 6) doses. Error bars show SEM values, and asterisks show significant changes compared with control (* *p* < 0.05; two-tailed Student’s *t*-test).

Changes in the delta and theta power were statistically analyzed in the entire segment, spanning from the time of injection until the end of the post-injection waking period, i.e., until the first sleep signs occurred (39 ± 3 min, range: 17–60 min). As delta showed a tendency to decrease in power over time and as the time course of such a delta decline varied from one experiment to the next, these segments were divided into two sections, the first immediately following the injection (labelled “A412-s1” in Figure 3, Figure 4 and Figure 5. and a subsequent period of lower delta activity (labelled “A412-s2” in Figure 3, Figure 4 and Figure 5) which lasted lasting the start of the upcoming sleep episode. The average lengths of these two periods were similar but showed high individual variations; the first lasting 19 ± 3 min (range: 5–40 min) and the second lasting 19 ± 2 min (range: 5–36 min). For the control, segments of active waking, i.e., when the rat showed similar behavior to the behavior after an injection, were selected, forming four sections of the 2-day-long recording: the last active waking episode before the injection (pre-inj ctrl, Figure 3, Figure 4 and Figure 5) and three analogous episodes post-injection, occurring at 6.5 ± 1.0, 11.3 ± 1.2, and 16.3 ± 0.6 h after the time of the injection. Peak power values in the delta (1–4 Hz) and theta (5–10 Hz) frequency bands were calculated in each segment, normalized to pre-injection power levels in individual experiments, averaged over the group and statistically tested compared with controls. 

A drug-induced increase in delta activity in the forebrain appeared in a dose- and region-dependent manner (Figure 3A). Strong delta enhancement was observed in the PFC after 5 mg/kg of A-412997; the delta power increased close to 100% relative to the control (187 ± 68%; *n* = 5, *p* = 0.08). The delta power also increased in the HC (Figure 3B) in the first segment (144 ± 34%) but was inconsistent across experiments and did not reach significance (*p* = 0.16). The effect of a lower dose (3 mg/kg) was weaker, and no significant changes were detected in either forebrain structure. The most drastic A-412997-induced changes in delta activity were observed in the RE; the average peak power increased by ~5-fold compared with the control (Figure 3C; 507 ± 290%; *p* = 0.03) and then dropped by the second period, to a still 2-fold increase (192 ± 77%; *p* = 0.19). A-412997 had no effect at the 3 mg/kg dose. No significant difference was found between the frontal and caudal RE electrodes (see Section 4) in any of the recordings, indicating that delta increased in the entire nucleus. The delta frequency remained stable (~2 Hz) after the injection.

The strong increase in delta activity was also associated with marked changes in the structure of pair-wise coherences within the PFC-RE-HC circuit. PFC-RE coherence increased after the administration of 5 mg/kg of A-412997 (from 0.55 ± 0.1 in control to 0.74 ± 0.06; *p* = 0.06) in the first half of the post-injection period (A412-s1) but returned to its original levels later as delta activity a showed progressive decline. In contrast, PFC-HC coherence decreased in A412-s1 (from 0.25 ± 0.06 to 0.18 ± 0.02; *p* = 0.03), while RE-HC did not change (0.19 ± 0.07 pre-injection and 0.20 ± 0.07; *p* = 0.44 in A412-s1). An injection of A-412997 at the lower dose (3 mg/kg) slightly decreased RE coherences in the delta range with both the PFC (from 0.51 to 0.49; *p* = 0.06) and HC (from 0.23 to 0.20; *p* = 0.02). PFC-HC coherence increased in the first half and decreased in the second half of the post-injection period, but the *p* values varied around the *p* = 0.05 level of significance, depending on variations in the control episodes (range: 0.31–0.34). Thus, the activation of D4R by A412997 promotes functional delta connectivity, particularly in the PFC-RE circuit.

### 2.3. Effect of Dopamine D4 Receptor Activation on Theta Activity 

Sustained waking activity after D4R activation was accompanied by significant increases in theta power in all three structures (Figure 4). In the HC and PFC, theta increased after A-412997 administration at either dose (Figure 4A,B), limited to the first period after the 5 mg/kg dose (134 ± 69%, *p* = 0.08 in the PFC and 180 ± 34%; *p* = 0.04 in HC) but lasting longer after the 3 mg/kg dose (Figure 4B). Dose-dependent changes only occurred in the RE (Figure 4C); A-412997 elicited a significant theta increase at 5 mg/kg (214 ± 289%, *p* = 0.004) but no significant change in the 3 mg/kg dose. The theta power showed large inter-individual variations in the PFC, and especially in the RE, potentially due to behavioral effects.

Prominent alterations were detected in the theta frequency (Figure 5A), in contrast with stable delta frequency (Figure 5B). Under control conditions, the theta frequency mostly appears between 6 and 7 Hz; it was 6.41 and 7.08 Hz in the two groups used for subsequent injections of 5 and 3 mg/kg of A-412997, respectively. The actual theta frequency also varied with the animal’s behavior in its home cage (speed of locomotion, attention, etc.). Within-experiment comparisons showed, however, that A-412977 induced a decrease in theta frequency in most experiments, observed in five out of six rats after a 3 mg/kg injection and in three out of five rats after a 5 mg/kg injection. On average, the theta frequency decreased in both groups, but significantly only after the 3 mg/kg drug injection (5.94 ± 0.26 Hz, *p* = 0.03, vs. 5.62 ± 0.35 Hz, *p* = 0.08 after 5 mg/kg A412997). 

Theta coupling weakened in the PFC-RE-HC circuit, as indicated by significant changes in several pairwise coherences. The RE-HC theta coherence decreased from 0.28 ± 0.1 in both test periods, but the decrease was only significant in A412-s2 (0.13 ± 0.09; *p* = 0.02) and not in the first period (A412-s1: 0.17 ± 0.08; *p* = 0.12). Slight decreases in theta coherence were further detected between the other signals as well, mostly in the second half of the reaction (PFC-HC: from 0.29 ± 0.01 to 0.23 ± 0.10; *p* = 0.08 and RE-HC: from 0.28 ± 0.10 to 0.17 ± 0.08; *p* = 0.12). No significant changes in theta coherences were seen between any of the signal pairs after 3 mg/kg of A412997. Thus, D4R activation exerts reciprocal effects on coherence, promoting delta coherence while reducing theta coherence, with the net effect being the facilitation of delta-associated connectivity in the PFC-RE-HC circuit during wakefulness.

## 3. Discussion

The present study used electrophysiological recordings in freely behaving rats to investigate the effects of D4R stimulation on slow oscillations during waking states, in which these rhythms play an essential role in interregional PFC-HC communications. We found that the systemic administration of the D4R agonist A-412997 drastically enhanced narrow-band delta oscillations in the PFC and RE. The effect was dose-dependent, being significant after a drug injection of 5 mg/kg but not after 3 mg/kg. In contrast, a moderate increase in theta power did not show region- or dose-dependence but appeared in all structures after both doses. Indeed, it lasted longer in the PFC and HC after the lower dose over the entire ~40 min post-injection wake period, until the first sleep signs appeared. The frequency of theta oscillations decreased in most experiments, and was significant over the group at the lower dose. In conjunction with the increased coherence of the delta oscillations, the overall effect of D4R stimulation was to preferentially enhance synchronous delta activity within the PFC-RE-HC circuit.

It is important to emphasize that the effect of D4R was observed on delta activity in waking, which is fundamentally different from delta in SWS. Both appear in the 1–4 Hz frequency range. However, slow waves during sleep, constantly varying in their lengths, produce a wide-band spectrum, while wake delta is represented by a narrow-band spectrum with well-defined sharp peaks. It appears during motor activity, primarily in the frontal cortex, coincident with theta oscillations in the HC and was shown to serve as a powerful mechanism of coupling between forebrain regions in a behavior-dependent manner (see e.g., [34,52,53]). Thus, our findings indicate that the D4R may contribute to cognitive processes, at least in part, through acting on wake delta oscillations.

The distinction between functional narrow-band delta oscillations and wide-band delta activity was emphasized in recent studies but requires further research. In urethane-anesthetized rodents, it was demonstrated that a PFC delta oscillation can be elicited via the same intervention as a theta rhythm [47]. The stimulation of the pontine nuclei, mimicking the ascending arousal signal from the brainstem, is known to terminate wide-band delta activity, modeling SWS, and to switch forebrain activity to narrow-band oscillations, theta in the HC [12,13,15,16,30,47] and delta in the PFC [30,47]. The D4R agonist A-412997 specifically enhanced this latter delta pattern in the urethane model [30]. A specific advantage of this model is that by suppressing gamma activity, slow oscillations are accessible for testing even after an injection of 10 mg/kg of A-412997, which in freely moving animals elicits abnormally high gamma activity [14], thus masking slow oscillations. In freely moving rats, the strength and character of gamma activity induced by the A-412997 at this dose were similar [14] to the aberrant gamma enhancement in rodent models of schizophrenia elicited via an NMDA-blockade [12,54,55,56,57,58]. At this concentration, it could provide a high level of background D4R activation upon which the effect of endogenous dopamine would be washed out, resulting in impaired performance and perhaps even schizophrenia-like cognitive deficits. Changes in network oscillations, i.e., abnormal gamma activation and disturbing the delta–theta balance in PFC-HC communication, may be important components of the neural mechanisms leading to this effect.

The behavioral effect of D4R agonists is, however, dose-dependent. Several studies showed that D4R agonists increased cognitive performance in low doses [59,60,61] but led to severe impairment after the injection of higher doses [61]. As for forebrain network activity, normal gamma enhancement is an important component of network activity which is necessary for numerous cognitive processes [62,63,64,65,66], and its modulations by slow oscillations were shown to be essential for interregional coupling [30,31]. In this study, we found a significant reorganization of the structure of theta–delta coupling in the PFC-RE-HC network after D4R activation by the agonist administered at the higher dose of 5 mg/kg, which was shown earlier to increase gamma power [14] but not to elicit aberrant gamma enhancement. We found a shift in the delta–theta balance to stronger delta coupling that might enhance performance in the components of memory tasks controlled by the PFC over HC-controlled spatiotemporal aspects [34,67,68]. The RE was found to play a key role in this control.

Further studies are needed to establish which D4R signaling mechanism is responsible for its effects on PFC-RE-HC delta and theta activity in wake states and to establish its importance in schizophrenia. In this regard, impaired methylation and underlying oxidative stress are well-established metabolic features of schizophrenia [25,69,70], both of which can contribute to impaired D4R-mediated phospholipid methylation, which has been proposed to modulate network oscillations to gamma frequency [14,25,29]. Moreover, high frequency gamma oscillations place a high demand on mitochondrial ATP production in parvalbumin-expressing interneurons, placing them at an increasing risk of oxidative stress from the production of reactive oxygen species [69,71,72,73,74]. An impairment of D4R-mediated PFC-HPC coupling via RE delta activity may thus be a relevant neurobiological mechanism in schizophrenia. 

Importantly, theta and delta rhythms also modulate local oscillations at frequencies higher (HFO; 80 Hz, >100 Hz [2,6]) and lower (beta; 15–30 Hz) than the gamma range (30–80 Hz), and their functional significance was demonstrated [2,46,48,49,50,51]. Jayachandran et al. [34] recently showed that the synchronization of beta bursts in the PFC, RE, and HC dominated memory trials which they managed to separate from theta dominance associated with running and thus obscuring other memory-related rhythms. They also showed that in this task, beta was not modulated by theta but rather the beta bursts appeared strongly coupled to delta phases. The origin of PFC-HC beta coupling was further tested via the optogenetic stimulation of the RE, which indeed increased beta activity in a task-dependent manner. In parallel to beta, optogenetic RE stimulation also increased delta activity and suppressed theta power and coherence. 

Finally, we want to point out several important limitations of the current study that will need to be considered in future research. The D4R agonist used in this study was administered via intraperitoneal injection, and thus the exact location of drug action remains unknown. D4Rs are widely distributed in the brain and in peripheral organs, such as the heart and kidney [75,76,77]. Since we only recorded brain activity in a carefully selected subset of structures, our studies obviously do not allow for the conclusion that the D4R effect was locally produced in or selective for the PFC-RE-HC circuit. D4Rs are found in all regions of the HC and in the PFC and other cortical regions, such as the frontal, parietal, and piriform cortex, as well as in non-cortical structures within the reticular thalamus, striatum and nucleus accumbens [78,79,80], and their localization even exceeds the localization of dopamine terminals [78]. On the cellular level, D4Rs are expressed by GABAergic interneurons, including parvalbumin-positive cells, which are essential for all network oscillations, and certain populations of pyramidal cells [78,80]. Analogous to the wide effect of the D4R agonist in our study, clozapine’s beneficial effects in schizophrenia may be achieved, in part, through D4-mediated GABA modulation, possibly implicating the disinhibition of excitatory transmission in multiple intrinsic cortical, thalamo-cortical and extrapyramidal pathways [80]. 

This uncertainty in the target structures may explain, at least in part, the prominent difference in the effects of D4R activation on theta rhythm in freely moving rats in this study versus the urethane anesthesia model we used earlier [30], i.e., the increased theta power in the former and the decreased theta power in the latter. A-412997 was administered through systemic injection in both studies, yet the target structures monitored in the two models were not identical. Under urethane anesthesia, theta is elicited via the stimulation of a well-defined ascending pathway from the brainstem, whereas in freely moving rats, theta is generated via a wider, highly interconnected network comprising the HC and cortical and subcortical structures to dynamically regulate the attributes of theta (e.g., frequency, amplitude and coherences) according to on-going behavior. Although adequate and widely used to study the specific mechanisms of network oscillations, the urethane model is commonly employed in parallel with studies on freely moving animals to achieve a meaningful interpretation of the data and to make valid conclusions regarding different levels of neural organization [30,81,82,83].

## 4. Materials and Methods

### 4.1. Animals

Male Sprague Dawley rats (3 months old, 300–350 g at the start of the experiment) were supplied by Charles River Laboratories, Wilmington, MA, USA. The animals were kept in the animal facility for at least 48 h before surgery and single-housed after the surgery under controlled lights with 12 h of light and 12 h in the dark, constant temperature and humidity and with freely accessible food and water ad libitum. All the experiments were performed in accordance with the animal protocol approved by the Beth Israel Deaconess Medical Center’s Institutional Animal Care and Use Committee (IACUC protocol #015–2015).

### 4.2. Drugs

The selective Dopamine D4 receptor agonist A-412997 used was supplied by Tocris (Abcam Inc., Cambridge, MA, USA) and dissolved to a concentration of 10 mg/mL. The drug was injected subcutaneously in doses of 3 mg/kg in the low-dose group and in doses of 5 mg/kg in the high dose group, and doses were calculated based on the weight of each rat; the average growth rate for the rats is 40 g/week. A-412997 was used due to its high selectivity and high affinity for both human and rat D4 receptors. The non-competitive NMDAR antagonist ketamine (Ketaject, Phoenix™, St. Joseph, MO, USA) was used with Xylazine for anesthesia during surgery and euthanasia before perfusion or decapitation.

### 4.3. Surgery

Survival surgeries were performed on 11 rats under anesthesia in sterile conditions. Prior to the surgery, all equipment was sterilized using dry heat in an autoclave (Columbus Dental), and gowns and sterile gloves were used throughout the whole procedure. Initial anesthesia was achieved by injecting a ketamine–xylazine mixture (30–40 mg/kg of ketamine and 5 mg/kg of xylazine) intraperitoneally. Full anesthesia was verified via no response to a pinching of the tail or paws. Supplementary ketamine injections (10% of initial dose) were given as necessary. The rat was fixed in a stereotaxic frame, and its body temperature was kept stable using an isothermal pad with heated water circulating. The site of incision and the surrounding area were shaved and disinfected using a povidone iodine solution (10%) and sterile cotton swabs. 

Stereotaxic coordinates were calculated relative to the bregma, according to the *Rat Brain Atlas of Paxinos*. Two stainless-steel screws were attached to the skull to act as a reference anterior to the frontal cortex and a ground reference above the cerebellum. A twisted double-wire electrode (an insulated stainless-steel wire of a 0.0005 inch diameter, PlasticsOne, Roanoke, VA, USA) was implanted in the dorsal and ventral HC (AP: −3.5; Lat: 2.2, D/V; −3.7 and −3.5 mm) and the PFC (AP: +3.2; Lat: −0.5, D/V: −5.1 mm). In the RE, electrodes were implanted at one (*n* = 3; AP: −2.5; Lat: 0.0, D/V: −7.0 mm) or at two different coordinates (*n* = 3) in the rostral (AP: −1.8; Lat: 0.0, D/V: −7.0 mm) and caudal (AP: −3.2; Lat: 0.0, D/V: −7.0 mm) aspects of the nucleus, with alternating single-wire and double-wire electrodes (tip separation < 1 mm) in each rat—single frontal and double caudal electrodes (*n* = 4) or double frontal and single caudal electrodes (*n* = 3). The PFC and HC electrodes were implanted on the right side of the skull, and the RE electrodes were implanted on the midline. After inserting each electrode, dental cement was applied to the skull to keep the electrodes in place. For EMG recording, Meloxicam analgesics (1 mg/kg of 5 mg/mL) was injected subcutaneously, and two multi-threaded electrodes with soft insulation were inserted into each side of the neck muscles. The electrodes were connected to a pair of six-channel connectors fixed on the skull with dental cement. An antibiotic gel was applied prior to suturing the incision, a Meloxicam analgesic (1 mg/kg of 5 mg/mL) was injected subcutaneously and the rat was observed until fully awake. A recovery time of at least one week was given before any recordings were conducted. After a few weeks, when all the recordings were completed, the rats were euthanized via a ketamine overdose.

### 4.4. The Identification of Electrode Position

The rats were transcardially perfused, first with phosphate-buffered saline for 5 min and then with a 10% buffered formalin solution (15 min). The extracted brain was stored in formalin at 4 °C for at least 7 days. Then, the formalin solution was exchanged for a sucrose solution (20%) for another day until the sucrose had completely penetrated the brain to lower the freezing point and thus minimize damage to the cells during cryosectioning. The locations of the electrodes were determined using three sample brains mounted on slides and evaluated under a microscope, and the brains were stored for future processing, using Cresyl Violet for staining if necessary. Before mounting, four drops of Permaslip (Alban Scientific Inc., Saint Louis, MO, USA) were applied to the slides before cover-slipping them with a cover glass (Brain Research Laboratories, division of Cambridge Intelligent Systems, Inc., Newton, MA, USA). The slides were left to dry and then viewed under a light microscope (Nikon ECLIPSE, E400, Suffolk, NY, USA) and compared with the brain atlas to verify the electrode positions.

### 4.5. Data Recording

For the recordings, the rats were placed in a recording box with food and water ad libitum and connected to an amplifier (A-M systems). First, the rats were left to accommodate to the recording box for 24 h while recordings were made on normal, undisturbed behavior. Next, recordings were made in conjunction with drug administration performed during the light period of the day (~6 h after lights were turned on). The rats were disconnected and injected with A-412997 subcutaneously in doses of 3 or 5 mg/kg, reconnected, and then continuously recorded for 24 h, including post-injection and control recordings which included segments of exploring, grooming, quiet waking, and naturally alternating sleep–wake states over both the light and dark periods of the day.

Local field potentials (LFPs) were recorded using a DASYLab 7.0 (Electronic Design, USA) and the stored ~.DDF-files were converted into into ~.SMR-files for processing using the Spike2 software package (version 7.00; Cambridge Electronic Devices, Cambridge, UK). The LFPs were filtered (low-pass filter < 18 Hz) and used to generate “sonograms”, (time–frequency plots, 0–15 Hz; see Figure 1 and Figure 2) to facilitate the observation of segments with prominent delta or theta spectral components. The LFP channels were viewed together with neck muscle EMGs to determine different states such as sleep–wake states, including SWS and REM sleep and quiet wake (QW) and active wake states (AW), using standard criteria for scoring. Thus, (1) minimal EMG activity was observed in a sleeping state and high EMG activity was observed in awake states; (2) during sleep, strong wide-band delta activity identifies SWS and high theta activity identifies REM sleep; (3) in waking, prominent theta activity identifies what is considered to be an AW state, while QW is associated with low theta activity.

### 4.6. Data and Statistical Analysis

Segments recorded after the injection of A-412997 were compared with data segments selected from the 24 h recordings before and the 24 h control period after injection. As the rats were awake after the injection, control data segments for comparison were selected during periods when the rats were awake. An effort was made to select longer-lasting awake segments with no visible artefacts. Five segments were analyzed for each rat. The first was recorded immediately after the injection and included a segment with visible alterations which were qualitatively obvious in the time–frequency plots. Its length varied between experiments and on average lasted 19 ± 3 min (range: 5–40 min). The second segment followed the first and lasted until the first sleep period (19 ± 2 min; range: 5–36 min). Four control waking segments 20–50 min in length were then selected, representing the last waking episode with motor activity before the injection and three episodes post-injection, occurring 6.5 ± 1.0, 11.3 ± 1.2, and 16.3 ± 0.6 h after the time of injection. 

Power spectral and coherence analyses were performed using the Spike2 program to determine the distribution of power and neuronal synchronization between different signals in these selected segments. Peak power values in the delta (1–4 Hz) and theta (5–10 Hz) frequency bands were found in each segment and normalized to the pre-injection control values in each experiment. Statistical analysis first verified no significant differences between the pre- and post-injection segments and then tested delta and theta power in the two test periods after injection against the average of all control segments. 

The coherence function was calculated for pairs of LFPs recorded from two spatially different areas, such as the PFC-HC, PFC-RE and HC-RE networks. The coherence value is between 0 and 1 where 1 is when two signals are completely phase-locked at a specific frequency and 0 is when there is no phase relation between two signals. For statistical comparisons, spectral power values within the delta (1–4 Hz) and theta (5–10 Hz) bands were calculated for each signal. The dominant peak was identified in these bands of the power spectra and the coherence values at that frequency. The power and coherence values were then averaged over the segments in the low-dose and high-dose groups, respectively, and statistically tested. The differences between the pre- and post-injection values of power and coherence were tested using a two-tailed Student’s t-test; the latter values were tested after a Fisher r-to-z transformation was performed to acquire z-scored values with normal distribution.

## 5. Conclusions

The present data demonstrate the involvement of D4R mechanisms in the reorganization of the structure of theta–delta coupling in the PFC-RE-HC network. In conjunction with our prior studies [14,30], this shows that the administration of a D4R agonist elicits a lasting awake state, enhanced gamma activity and preferentially enhanced synchronous delta activity over theta coupling within the PFC-RE-HC circuit. The modulation of fast beta and gamma network oscillations via slow delta and theta rhythms play a key role in behavior-dependent interregional cortico-hippocampal coupling. Thus, our findings indicate that the D4R may contribute to cognitive processes, at least in part, through acting on wake delta oscillations.

## Data Availability

Not applicable.
